# Speckle lithography for fabricating Gaussian, quasi-random 2D structures and black silicon structures

**DOI:** 10.1038/srep18452

**Published:** 2015-12-18

**Authors:** Jayachandra Bingi, Vadakke Matham Murukeshan

**Affiliations:** 1 Center for Optical and Laser Engineering, School of Mechanical and Aerospace Engineering, Nanyang technological University, Singapore 639798

## Abstract

Laser speckle pattern is a granular structure formed due to random coherent wavelet interference and generally considered as noise in optical systems including photolithography. Contrary to this, in this paper, we use the speckle pattern to generate predictable and controlled Gaussian random structures and quasi-random structures photo-lithographically. The random structures made using this proposed speckle lithography technique are quantified based on speckle statistics, radial distribution function (RDF) and fast Fourier transform (FFT). The control over the speckle size, density and speckle clustering facilitates the successful fabrication of black silicon with different surface structures. The controllability and tunability of randomness makes this technique a robust method for fabricating predictable 2D Gaussian random structures and black silicon structures. These structures can enhance the light trapping significantly in solar cells and hence enable improved energy harvesting. Further, this technique can enable efficient fabrication of disordered photonic structures and random media based devices.

Disordered photonics has made significant contributions to the field of science and engineering in the recent past with potential applications ranging from imaging, random lasers to solar energy harvesting^1^,^2^. Most of the natural systems in our daily life such as human tissue, blood cell, milk colloid, clouds, marble stone etc., strongly scatter light due to their structural disorder^3^. The surface phenomena such as higher brightness^2^, superhydrophobicity, superlyophobicity, and super oleophobicity also stem from the structural randomness of the surface^4^. Disordered systems host interesting optical phenomena such as coherent backscattering^5^, Anderson localization^6^, super and hyper diffusion^7^, wave focusing^8^, random lasing^9^, enhanced energy storage^10^ etc. On the other hand, quasi-random structures are shown to be a new class of materials for broadband photon control. Quasi-random and Gaussian random structures (2D random structure) are proposed as good candidate for energy harvesting^11^,^12^,^13^,^14^. All physical phenomena in a random medium are demonstrated in arbitrary random structures that are fabricated by different methods such as removing the particles from random positions in photonic crystal^15^, self-assembly of particles^9^, deep reactive ion etching^16^, chemical vapor deposition^17^, introducing particles into polymer matrix^18^, and femtosecond laser focusing to create randomly distributed voids^19^ and electron lithography for small area fabrication^20^. More or less all fabrication methods suffer from lack of reproducibility, control and limitations attributed to wide area fabrication. Hence, random medium is least exploited for device fabrication in spite of all its advantages. However, a fabrication strategy that results in predictable, tunable and reproducible random and quasi-random structures will open up new possibilities with which we can tailor them towards future device applications. In this paper, we propose and demonstrate a robust and novel method, named as speckle lithography, to fabricate 2D random and quasi-random structures.

The lithography has been emerged as a widely used and standard technique for micro and nano structure fabrication. Significant number of strategies have been used in this technique such as photo-lithography, nano imprint lithography, electron beam lithography, soft lithography and dip pen lithography^21^,^22^. Recently several non-conventional lithography techniques such as crack assisted photo-lithography^23^, nanomotor lithography^24^ and plasmon assisted photo-lithography^25^,^26^ are implemented successfully. Generally, the laser generated speckles in these photo-lithographic techniques reduce the pattern contrast and quality, so speckles are considered as noise^27^.

Lithography approach such as nanoimprint method can be adopted for fabricating random and quasi-random structures. However, it is a multi-step process and depends on many parameters to achieve the desired structures^11^,^14^,^28^. In the case of self-assembly approaches, they need highly controlled chemical ambience and related protocol to achieve the desired reproducibility and controlled feature writing. Both the above mentioned approaches have limitations compared to the proposed speckle lithography, which is a noncontact and whole field method that offers tunability in writing features of different sizes and randomness.

Further, the laser generated speckle patterns are the natural random intensity patterns (distributions) which follow the Gaussian statistics. The probability distribution of speckle intensity pattern is


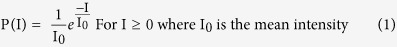


The speckle size distribution is calculated from spatial intensity correlation^29^. Further, speckle size, depth and density are well controllable by simple optical configuration. And the speckle cluster generation is reported to be possible when the speckle beam travel through multiple apertures arranged in a particular geometry^29^. These features of the speckle patterns prompted us to use speckle pattern itself for photo-lithography, which efficiently generates the predictable, tunable and well controlled 2D Gaussian random and quasi-random structures with the proposed concepts and methodology. Based on, the speckle statistics, radial distribution function and fast Fourier transform concepts; we also propose quantifying criteria for the randomness of structure in a specified area. Further, the black silicon random structures are fabricated in a controlled fashion by controlling the size and density of speckles which demonstrates the potential of this technique.

## Results and Discussion

### Fabrication and quantifying the Gaussian random structure by speckle lithography

**Figure 1:**
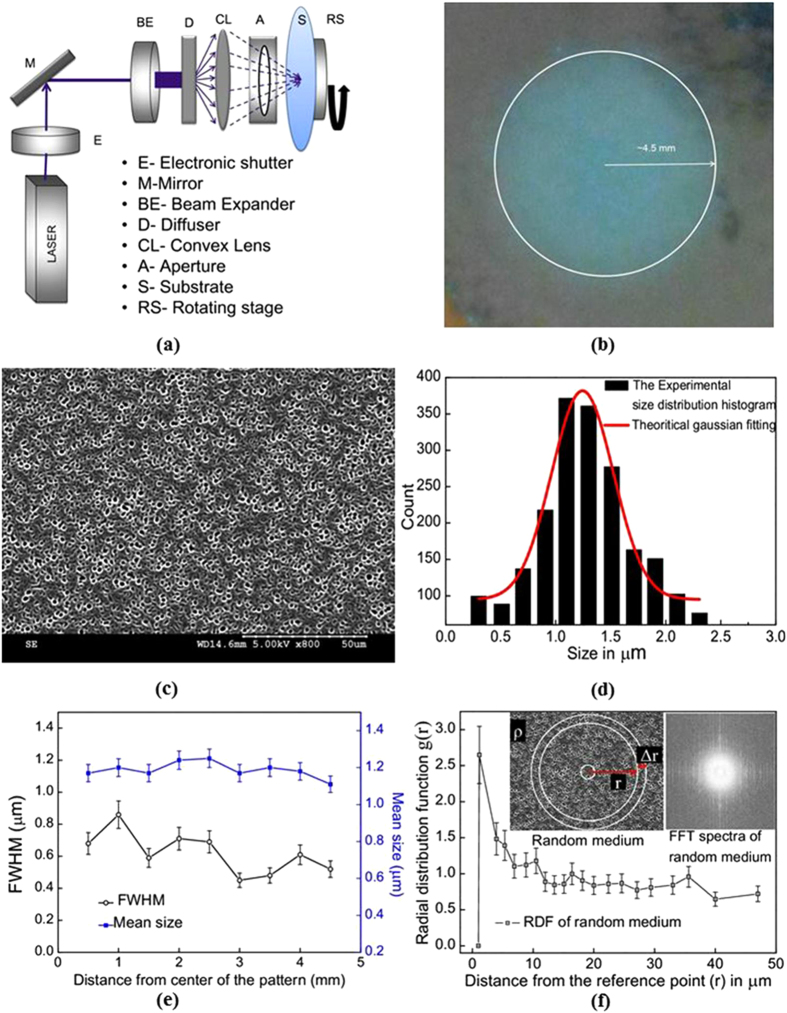
(**a**) The speckle photolithography experimental setup, (**b**) image of pattern recorded on AZ 7220 photoresist on silicon substrate, (**c**) The SEM image of the speckle pattern on photoresist, (**d**) The speckle spot size distribution calculated from SEM image, (**e**) The mean size of the speckle spot (Blue curve) and the FWHM of distribution at different distances from center of the pattern, (**f**) The plot of RDF for random medium, inset shows the SEM image explaining the RDF procedure and the FFT spectrum (SEM images are taken and figures are composed by Jayachandra Bingi)

The basic procedure for the proposed speckle lithography technique is to generate the speckle pattern with certain mean speckle size and project them onto the substrate coated with a photoresist. Figure 1a shows the experimental set up used for speckle lithography, where the laser beam of 354 nm is directed towards the diffuser (**D**, a ground glass plate) through an electronic shutter (**E**) and a beam expander (**BE**). The subjective speckles generated at the diffuser (D) are collected by a convex lens (CL). Further, these speckles are projected onto the substrate (S) through an aperture (A). The exposure time and the beam diameter are adjusted by electronic shutter (E) and beam expander (BE) respectively. According to the basic speckle theory, speckle width and the depth are governed by^30^


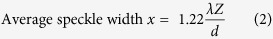



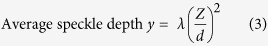


where Z is the distance from lens (CL) to substrate(S), *d* is diameter of the circular aperture (A) and λ is the wavelength. Here Z depends on the diffuser to the lens (D-CL) distance, even though the Z distance is related to the D-CL distance by an equation reported elsewhere^30^. Here we use only the optimized distances (D-CL and Z) for the well-defined pattern to be printed on the photoresist. The experimental parameters such as laser beam diameter, exposure time, lens diameter, focal length, D-CL distance, Z (CL-S distance), and diameter of aperture are chosen as 2 mm, 500 ms (milliseconds), 25 mm, 25 mm, 45 mm, 35 mm and 15 mm, respectively. Among these the laser beam diameter, D-CL distance and Z (CL-S distance) are variable parameters that are varied according to the speckle size and density requirements.

The speckle pattern recorded on the photoresist is shown in Fig.1b, which is a circular pattern of 4.5 mm radius. Figure 1c, the scanning electron microscope (SEM) image of the pattern, is showing the random distribution of spots created by speckles on the photoresist. The speckle spot size distribution is calculated from the SEM image using the *Image j* software. Figure 1d shows the experimentally calculated histogram of size distribution and its Gaussian fit. The good agreement between the experimental and theoretical distributions confirms that it is a Gaussian random structure with the mean spot size and size distribution calculated as 1.24 (±0. 018) μm and 0.66 μm, respectively.

However, there must be quantifying criteria in order to confirm the randomness in a structure. Any physical random medium can have randomness with respect to the size and the position of particles. In the present 2D Gaussian random medium, the full width at half maximum (FWHM) of the Gaussian size distribution curve represents the randomness in size. Figure 1e shows the mean size and FWHM variation as a function of distance from center to periphery of the pattern (Fig. 1b). It can be noticed that the mean size is almost constant with insignificantly small variation with a value of 0.048 μm, approximately. However, the size randomness (FWHM) fluctuates between 0.5 μm – 0.9 μm. On the other hand, the positional randomness of the medium depends on the positional distribution (random or quasi-random) and the packing density of the structure. To quantify these, we consider the 2D radial distribution function (RDF), **g(r),** which is defined as the density variation with distance from an arbitrary reference point in a specified region. The RDF is given by^31^


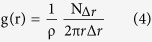


where **ρ** is the average density of system, **N**_**Δr**_ is number of particles in an annular shell with width **Δr** and at distance **r** from an arbitrary reference point as shown in the inset of Fig. 1f. As shown in the SEM image (inset of Fig. 1f), we consider a reference point at the center of the cell and assume the concentric 2D shells with 5 μm width **Δr**, at different distances **r** from the center. The **g(r)** (RDF) values are calculated at different distances by considering static system (time invariant 2D structure). Figure 1f shows the plot of ***g(r)*** Vs ***r,*** where the RDF increases to maximum value near the reference point and further reducing rapidly towards unity. There are no well-defined peaks along the radial distance which indicates the absence of the periodicity^31^,^32^ and hence the existence of randomness in the structure. Further, though the randomness can efficiently be indicated by the RDF, it is unable to give the information on short range or very short range periodicities in large area structures. These short range periodicities are probed in this work by taking the fast Fourier transform (FFT) of the surface (as there are 2D structures). Basically, FFT spectrum of an image represents the image in frequency domain. The inset of Fig. 1f also shows the FFT spectra of random medium surface, which is a random distribution of data points in frequency domain. This does not show any specific frequency distribution that is generally attributed to short range periodicity which confirms the absence of short range periodicities. Hence, the analysis in terms of the Gaussian size distribution, RDF and FFT concepts revealed that the structure under consideration (Fig. 1c) is a randomly packed structure (confirmed by RDF curve and FFT) with an average RDF (local packing density factor) of 1.036, a mean spot size of 1.19 μm, and an average size randomness (spread) of 0.576 μm.

### Tunability of speckle spot size and density

**Figure 2:**
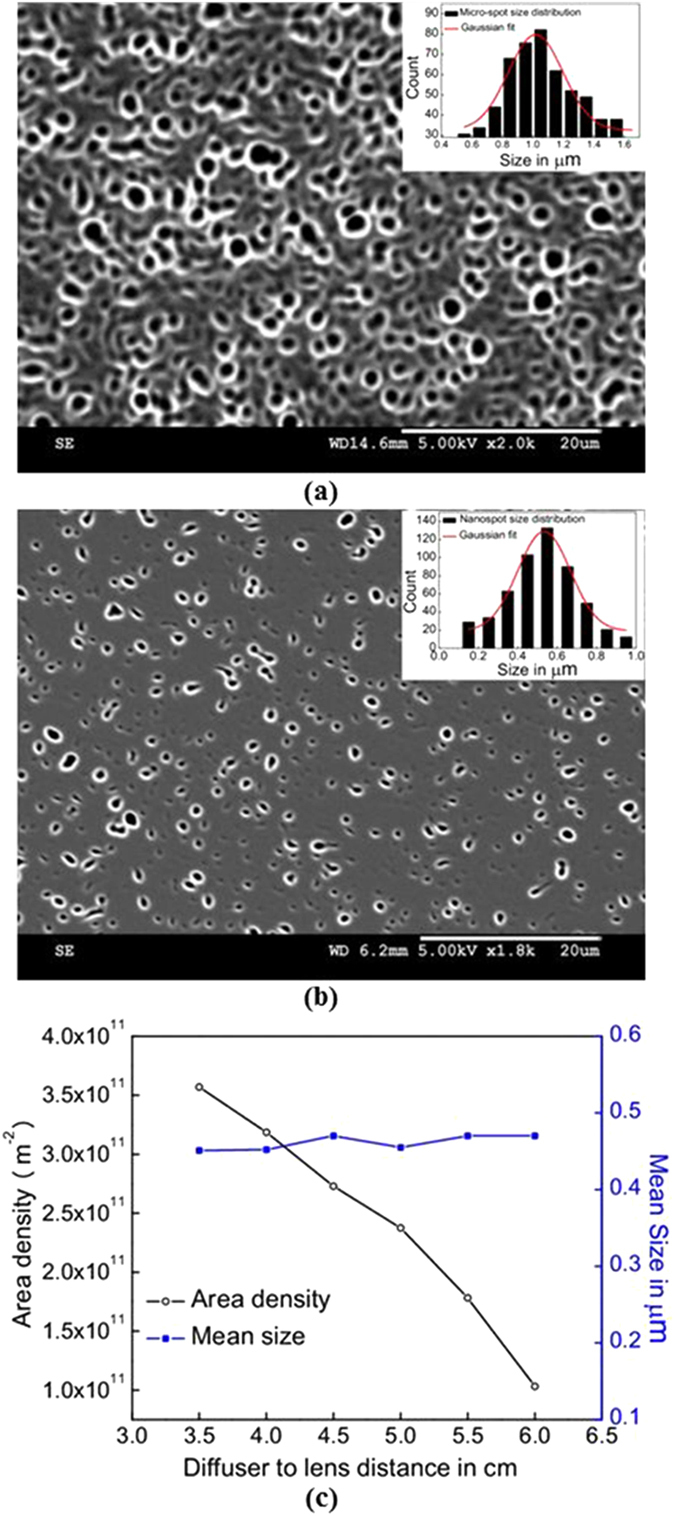
(**a,b**) the micro and nano patterns recorded with beam diameter 2 mm and 12 mm respectively, (**c**) The plot shows the area density and spot size as a function of D-CL separation.

Along with the randomness, the speckle size tunability is crucial to control the wavelength dependent scattering properties of the random structure. The tunability in speckle size is achieved by varying the area of illumination (incident beam diameter) on the diffuser (***D***). Figure 2a,b show the two patterns recorded at beam diameters 2 mm and 12 mm. The speckle size after the diffuser is inversely proportional to the illuminating area which indicates that divergent beam illumination generates finer speckles. The speckle size distributions are shown in the inset of Fig. 2a,b where the average mean size is reduced from 1.02 μm to 0.53 μm.

Besides the size tunability, the density of the speckle spots (here “speckle spot” refers spot created by speckles on the substrate medium) in the pattern can also be made tunable, which is very important to fabricate strong scattering 2D random medium. This can be done by varying the D-CL distance and by keeping the exposure area (diameter) on substrate (S) constant. The speckle beam generated at the diffuser diverges at greater angles compared to an ordinary laser beam. Hence at smaller D-CL distances, the speckle beam incident onto the convex lens (CL) with smaller incident area and higher density of speckles projected onto the substrate (S). Increase in D-CL distance results in the larger incident area and hence the speckle density incident on convex lens (CL) reduces as they are inversely proportional. Figure 2c shows how the area density varies with the D-CL distance. It can be seen that the area density reduced gradually from 3.6 × 10^11^−1 × 10^11^ when the D-CL distance was changed from 3.5 cm to 6 cm. Figure 2c (blue curve) the spot size is nearly constant in patterns recorded at different D-CL separations which confirms the ability of the technique to fabricate random media of different density with constant mean size.

### Speckle clusters for fabrication of quasi-random structures

**Figure 3:**
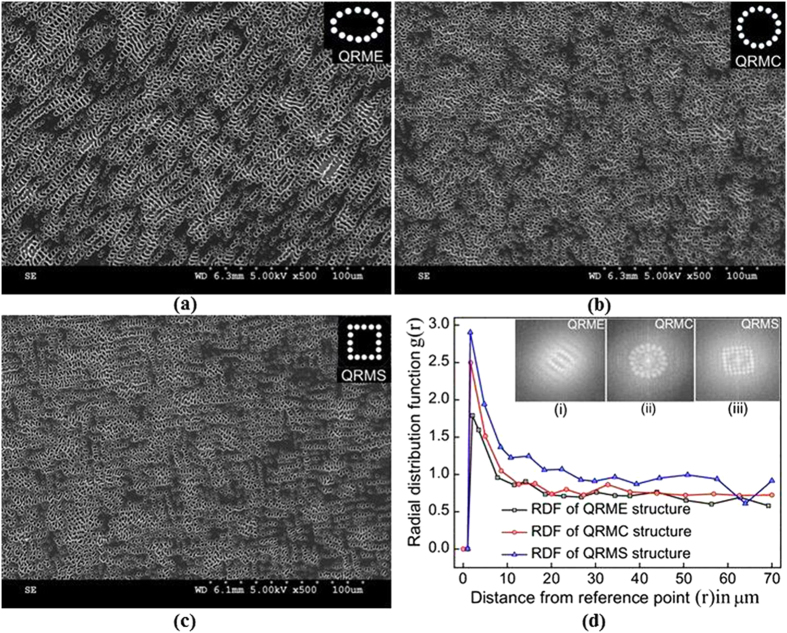
The SEM images of quasi-random structures obtained by using (a) Elliptical, (b) Square, (c) Circular pupils, (d) The RDF plots of the structures QRME, QRMC and QRMS, inset shows the FFT of the same.(SEM images are taken and figures are composed by Jayachandra Bingi).

Further, the proposed technique is extended to fabricate the quasi-random structures based on the concept of speckle clustering. The subjective speckles travelling through multiple aperture pupil form the speckle clusters. This is due to the superposition of the different speckle patterns emanating through different apertures in the pupil. Depending on the pupil geometry, variety of clusters which forms the quasi-random patterns can be attained. This is done by replacing the circular aperture with multiple aperture pupil. Figure 3a–c show the quasi-random patterns resulted upon using elliptical (QRME), circular (QRMC) and square (QRMS) pupils (shown in inset) respectively. These structures are similar to the clusters reported in the literature^32^. Here, the speckle size and separation could be tuned by pupil geometry, incident beam diameter, and D-CL distances same as in the case of the random medium.

Figure 3d shows the plot of RDF for different quasi random structures QRME, QRMC and QRMS. As stated previously, the RDF curves of these structures are similar to the random medium. This indicates the dominant randomness in these structures. The maximum RDF value near the reference point is high for QRMS (~2.9) compared to others, which is the signature of the higher packing fraction of this structure. The inset of Fig. 3d shows the FFT spectra of these structures where the well-defined pattern in FFT spectra clearly distinguish these structures, which confirms the existence of short range periodicities. Hence, RDF and FFT spectra reveal that the structures are the quasi-random structures with short range periodicities.

From the SEM images (Fig. 3a–c) and the FFT spectra of the quasi-random medium surface, it is clear that the speckle clusters are showing the shape correlation with the geometry of pupil. In this way new and variety of structures can be generated, as a random distribution of an arbitrary cluster of specific geometry, by selecting the pupil accordingly. According to the recent reports^11^,^14^ the quasi-random structures play a significant role in light trapping. The variety of quasi-random structures realized through this method opens up new possibilities for light trapping in Si structures.

### Fabrication of black silicon in controlled fashion

**Figure 4:**
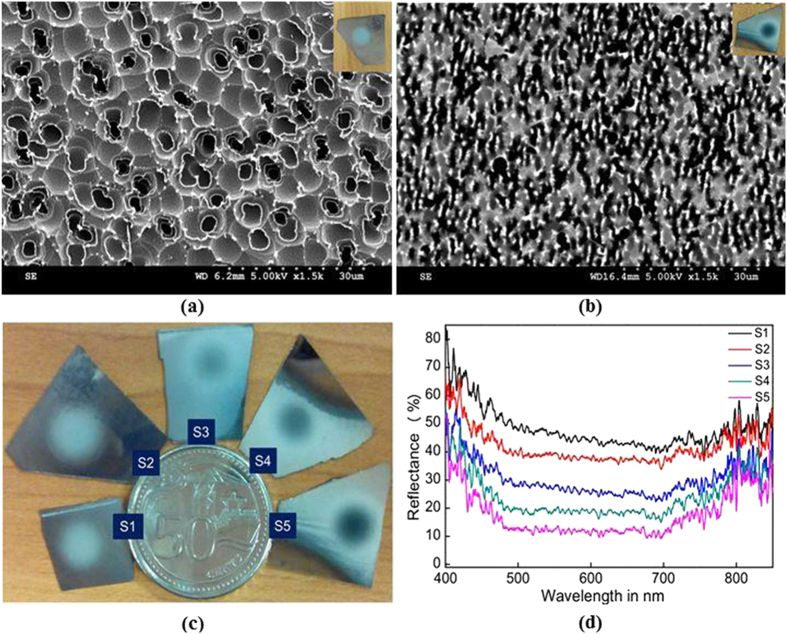
(**a,b**) SEM images of dry etched Si with low and higher density of speckle spots respectively, (**c,d**) The image of the speckle lithographically made patterns on Si substrate and their respective reflections. (SEM images are taken and figures are composed by Jayachandra Bingi).

The potential of this speckle lithography technique is demonstrated by fabricating the black silicon in a well-controlled fashion, which is very significant in solar energy harvesting and IR photonics^33^. The random patterns with different speckle densities, ~ 0.7 × 10^10^, 0.9 × 10^10^, 0.9 × 10^11^, 1.7 × 10^11^ and 2.5 × 10^11^ (named as S1, S2, S3, S4, and S5) are fabricated on the photoresist coated on Si followed by dry etching. The D-CL distances are varied from 40 mm to 60 mm in steps of 5 mm for making samples S1-S5 respectively. Figure 4a,b show the lower (S1−0.7 × 10^10^) and higher (S5- 2.5 × 10^11^)spot density samples after etching, where the speckle density clearly made a huge difference in their reflection properties after etching (S2 and S5 Substrates are shown in inset).

The Fig. 4c is the image of etched silicon substrates with increasing speckle density 0.7 × 10^10^ −2.5 × 10^11^ (Sample S1-S5) clearly showing the reduced reflection at higher speckle densities. Figure 4d is the reflections from the substrates patterned with different speckle densities where the structure with maximum speckle spot density (S5) reflecting only 10% of incident light. At the same time the samples S1 and S2 are showing higher reflection than a plane Si substrate which generally shows the reflectance ~20–30%. This higher reflectance of S1 and S2 is due to the surface roughness introduced by etching process which is evident from Fig 4a. More clearly, when the speckle pattern is projected onto the substrate, the speckles with power density beyond the threshold burn the photoresist and makes well defined spots. Speckle that contains power density lower than the threshold impact the photoresist partially and make the whole surface uneven. Hence, the etching process results in the uneven surface. This uneven rough surface is dominant compared to speckle spots in samples S1 and S2 (Fig. 4a).

This random texture over the sample surface has a curved feature with an average size of the order of microns. Further, the plane of incidence changes point by point in this random texture having curved surface features. In the case of ordinary silicon, the surface is flat and the plane of incidence is same at all points. Hence the curved features of surface random texture and change in plane of incidence could eventually be leading to high surface reflectance. This makes the pattern to appear brighter than the normal Si substrate. In the case of S3, S4 and S5 the speckle spot density is higher so that the reflection is going down. Evidently sample S5 appear as porous after etching because of higher speckle density where the reflection is reduced to <10%, hence creating a black Si. This proposed procedure can produce the black silicon with random and quasi-random structures which can enable efficient light trapping, harvesting and expected to possess better electrical properties. It is envisaged that such structures are highly desirable for better efficiencies in solar cells and future research will be in this direction.

The important aspect to be noted in this case is the change in spot size and surface morphology after etching. Generally, after the etching process, the spot sizes are observed to be wider than that of photoresist patterns, which is due to the weak etching resistance of photoresist at spot edges. Secondly, the surface also undergoes etching when the etching process is completed. This changes the surface morphology which again depends on the thickness of photoresist and its etching resistance. Hence, the choice of photoresist and its thickness are the important factors to get control over surface morphology.

Further, the resolution or the feature size that we obtain in this method, is a function of the wavelength, D-CL distance, Z (CL-S distance) and beam diameter (with the constant exposure area). We could experimentally achieve the lowest mean size (Up to now) of 0.47 μm with the wavelength 354 nm and beam diameter >12 mm (Fig. 2c). The black silicon is realized in literature with the surface features having size in the order of micron and submicron^34^,^35^,^36^,^37^. This is easily realized by the proposed speckle method also with comparatively less reflection (<10%). The speckle spot size can be further reduced by adopting the same method with shorter wavelengths such as 256 nm and 190 nm which can result in sub-micron to nanoscale size patterns. Besides the feature size, the surface structure affect the properties of black silicon^38^, hence the ability to fabricate quasi-random structures is clearly an advantage of this method.

In conclusion, a novel concept and methodology named as speckle lithography is proposed and demonstrated as a method for fabricating predictable and tunable Gaussian random and quasi-random structures. Based on the Gaussian statistics and random packing fraction concepts, the randomness of the structure is quantified by speckle statistics, radial distribution function (RDF) and Fast Fourier transform (FFT). The tunability of speckle spot size and separation is demonstrated by varying the experimental parameters such as D-CL distance and incident beam diameter on diffuser (Area of illumination). The versatility of this speckle lithography technique is in its ability to fabricate well controlled and tunable random structures which is demonstrated by fabricating the black silicon in a controlled manner. Further, this developed speckle lithography can open up the possibility for fabricating and realizing full-fledged random media based devices such as new generation solar cells with improved efficiency, optical analogue of electronic devices and sensors.

## Materials and Methods

### The substrate preparation and development

The experiments are performed on the silicon substrate coated with the AZ7220 positive photoresist. The Si substrate is treated with hexamethyldisilazane (HMDS) priming for 1 minute, followed by spin coating of photoresist at 5000 rpm for 30 seconds. The thickness of the photoresist is maintained at 1.2 μm–1.5 μm. After the photo exposure, the substrate is developed with MIF 826 developer for 1 min. For higher density speckle pattern, 30 seconds is sufficient to get the good pattern followed by a thorough rinsing. The developed substrates are etched for 10 minutes in reactive ion etching unit (RIE, Alcatel) with expected depth 10- 15 μm. The reactive ion etching is done by loading the substrates in the RIE Alcatel, STS ICP multiplex system, the parameters used in the present process are as follows

Machine: STS ICP multiplex system

Gas: SF_6_ 100 sccm, O_2_ 13 sccm, C_4_F_8_ 130 sccm

Cycle time: Etching 8 seconds and passivation 5 seconds (Starting with passivation)

Pressure: 5 mtorr

Power: coil power 800 W, platen power 10–12 W, multiple 10.

Etch rate: ~1 μm/1min (as the feature size is small)

The approximate depth is 10–15 μm depending on the machine and etching rate.

### Reflectance Measurements

**Figure 5:**
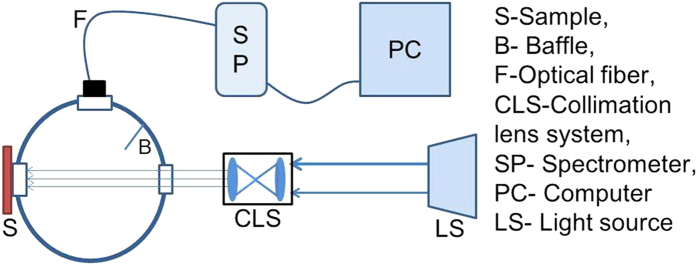
The reflectance measurement setup using an integrating sphere connected to spectrometer. The samples are exposed to broadband light source. (SEM images are taken and figures are composed by Jayachandra Bingi).

A broadband source (Edmund Optics, MI-150) and two lenses (Thorlabs, LB1761-A and LB1471-A) are used to produce the collimated white light. The setup in Fig. 5 measures R(λ), where the reference is a 99% reflectance standard (Labsphere, SRS-99-010). The reflected light travels through the optical fiber (Ocean Optics, QP400-1-VIS-NIR) and is detected by the spectrometer (Ocean Optics, USB4000) with SpectraSuite® software (Ocean Optics).

### Speckle spot density tuning

**Figure 6:**
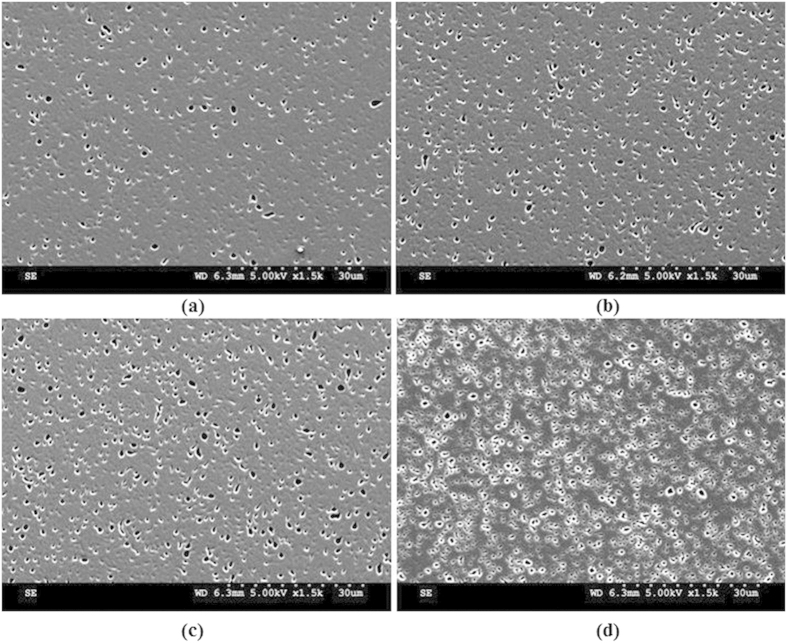
The SEM images of patterns with different area densities (a) ~0.7 × 1011, (b) ~1.3 × 1011, (c) ~1.9 × 1011, (d) ~3.1 × 1011 at 6 cm, 5.5 cm, 5 cm and 4 cm D-CL distances respectively. (SEM images are taken and figures are composed by Jayachandra Bingi).

The density of spots is not limited to what is shown in the SEM image shown in the manuscript alone. It can be widely tuned by adjusting the D-CL distance and CL-S distances accordingly and by keeping the exposure area constant (~9 mm). Figure 6 shows the SEM images of patterns with different area densities (a) ~0.7 × 10^11^ m^−2^, (b) ~1.3 × 10^11^ m^−2^, (c) ~1.9 × 10^11^ m^−2^, (d) ~3.1 × 10^11^ m^−2^ corresponds to different D-CL distances of 6 cm, 5.5 cm, 5 cm and 4 cm, respectively. Hence, this demonstrates the tunability of spot density using this method.

## Additional Information

**How to cite this article**: Bingi, J. and Murukeshan, V.M. Speckle lithography for fabricating Gaussian, quasi-random 2D structures and black silicon structures. *Sci. Rep.* **5**, 18452; doi: 10.1038/srep18452 (2015).
